# Inadequacy of coronary calcium scoring in evaluating coronary artery disease: A call to shifting to high-resolution CT coronary imaging

**DOI:** 10.1016/j.ijcrp.2025.200476

**Published:** 2025-07-18

**Authors:** Jonathan Mokhtar, Mohammad Albaree, Virginia Battistin, Mohamed Asbaita, Fatemeh Akbarpoor, Jeyaseelan Lakshmanan, Hassan El-Tamimi

**Affiliations:** a College of Medicine, Mohammed Bin Rashid University of Medicine and Health Sciences. Dubai Healthcare City, Dubai Health, Dubai, United Arab Emirates; b Mediclinic Parkview Hospital, Dubai, United Arab Emirates

## Abstract

**Background and aims:**

Coronary artery calcium (CAC) scoring is an increasingly adopted, non-invasive modality for assessing coronary artery disease (CAD). However, its diagnostic reliability in comparison to invasive coronary angiography (ICA) remains controversial. This study evaluated the diagnostic performance of CAC scoring in predicting CAD using ICA as the reference.

**Methods:**

Adults who underwent both coronary computed tomography angiography (CCTA) with CAC scoring and ICA within a three-month interval were retrospectively analyzed between 2018 and 2024. Obstructive CAD was defined as ≥ 50% stenosis on ICA. Patients were stratified by CAC scores: 0 (group 1), 1–399 (group 2), and ≥400 (group 3). Chi-square analysis was utilized to assess the differences in CAC scores compared to ICA. The sensitivity, specificity, positive predictive value (PPV), and negative predictive value (NPV) of CAC against ICA were all calculated using R version 4.4.0.

**Results:**

Among 110 patients (mean age 53 ± 10; 86.4% males), obstructive CAD was found in 25% of patients in group 1, 56% of patients in group 2, and 79% of patients in group 3 (χ^2^ = 14.21, *p* < 0.001). CAC demonstrated a sensitivity of 91.2%, specificity of 63.2%, a PPV of 92.2%, and an NPV of 60%.

**Conclusion:**

While a CAC score of 400 or higher strongly predicts significant CAD, scores of zero or intermediate values fail to exclude obstructive disease reliably. These findings reaffirm that CAC scoring is a useful stratification tool but should be interpreted with caution, particularly in high-risk patients, and confirmed with ICA when appropriate.

## Highlights

• CAC exhibits a high sensitivity and PPV, but low specificity and NPV.
• CAC score of 0 missed 25 % of obstructive CAD – a zero score doesn't mean zero risk.
• CAC score ≥400 strongly predicts true coronary artery stenosis.

## Graphical abstract

**Figure:**
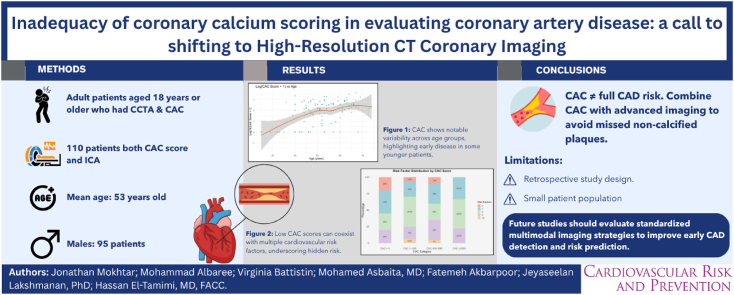

## 1 Introduction

Coronary artery disease (CAD) remains a leading cause of morbidity and mortality worldwide, necessitating accurate and efficient diagnostic methods for early detection and management. Invasive Coronary Angiography (ICA) is the gold standard for diagnosing significant CAD, providing direct visualization of coronary artery stenosis [1,2]. However, due to its invasive nature, associated risks, and considerable cost, there has been growing interest in non-invasive imaging modalities that can accurately identify and stratify at-risk patients while reducing unnecessary interventions [3,4].

Coronary artery calcium (CAC) scoring, derived from non-contrast computed tomography (CT) scans, quantifies the burden of calcified plaque and serves as a surrogate marker for overall atherosclerotic burden [5,6]. Numerous studies have shown that a CAC score of zero is associated with a very low probability of obstructive CAD [7]. Although CAC scoring is not highly specific for obstructive lesions, its high sensitivity makes it a powerful tool for exclusion [8]. In asymptomatic individuals and low-to-intermediate-risk patients, negative predictive values have been reported to approach 95–100% in some cohorts [7,[9], [10], [11]].

Biologically, coronary calcification reflects an active inflammatory and osteogenic process within the vascular wall [12]. Two histological types of calcifications have been described: intimal calcification, commonly associated with atherosclerotic plaques, and medial calcification, which is typically linked to systemic conditions such as diabetes and chronic kidney disease [12,13]. CAC scoring does not differentiate between these two entities, as it provides a cumulative calcium burden without identifying plaque composition or vulnerability [14]. It is most commonly quantified using the Agatston method, which incorporates both the lesion area and peak CT attenuation to estimate total calcium burden [15].

CAC scoring has become a valuable tool in cardiovascular risk stratification, widely used to guide statin initiation, refine pre-test probability, and inform the need for further diagnostic testing such as coronary computed tomography angiography (CCTA) [7,11,16]. Although CCTA evaluates stenosis severity and plaque morphology, the CAC score is derived from the preceding non-contrast phase [17,18]. The blooming artifact from partial volume averaging of densely calcified lesions can lead to overestimation of stenosis and affect diagnostic accuracy in heavily calcified vessels [19]. Nonetheless, CAC remains a distinct and complementary modality, particularly useful in primary prevention [20].

In this retrospective observational study, we evaluate the diagnostic accuracy of CAC scoring in detecting significant CAD using ICA as the reference standard. This study aims to determine whether CAC can reliably identify patients with obstructive CAD and reduce reliance on invasive procedures in appropriately selected populations.

## 2 Methodology

### 2.1 Study design

This retrospective observational cohort study was designed to compare the performance of non-invasive imaging techniques (CAC) to ICA, aiming to determine whether they could reliably reduce the need for unnecessary invasive procedures. The methods and results were reported in accordance with the Strengthening of the Reporting of Observational Studies in Epidemiology (STROBE) guidelines [21].

The study was conducted in accordance with institutional ethical guidelines and adhered to the Declaration of Helsinki. Ethical approval was granted from the Mediclinic Middle East Research Ethics Committee (Ref#: MCME.CR.373.MPAR.2024) and the Dubai Scientific Research Ethics Committee (Ref#: DSREC-03/2025_26).

### 2.2 Setting

The study was conducted at Mediclinic Parkview Hospital, Dubai, United Arab Emirates, a tertiary healthcare center equipped with high-resolution 256-slice multi-detector computed tomography scanners and a dedicated cardiac catheterization laboratory. The single-center design minimized data inconsistencies and inter-observer variability.

### 2.3 Participants

#### 2.3.1 Study population

The study population consisted of all adult patients older than 18 years who underwent both CAC scoring and ICA within a three-month interval at Mediclinic Parkview Hospital between October 2018 and October 2024.

#### 2.3.2 Eligibility criteria

Patients who met the inclusion criteria included: 1) patients 18 years or older; 2) had undergone a diagnostic CCTA with CAC scoring; and 3) had a follow-up diagnostic ICA within a time interval of less than three months.

Patients were excluded if they: 1) required emergency ICA due to acute coronary syndrome (ACS); 2) had previously diagnosed CAD; 3) had the ICA performed more than three months after CCTA; 4) had a previous percutaneous coronary intervention (PCI) or coronary artery bypass graft (CABG) surgery.

#### 2.3.3 Method of study subjects’ identification

Patients were identified through radiological and catheterization lab data within the hospital's electronic medical record (EMR) system. Radiological and procedural data were cross-matched to identify individuals who had undergone both CCTA with CAC scoring and ICA within the study period (≤ 3 months). Eligible patients were assigned anonymized unique identifiers to maintain confidentiality and ensure compliance with institutional data protection policies.

### 2.4 Variables

The primary variables included: 1) CAC scores recorded following the Agatston scoring method; 2) coronary stenosis severity assessed by CCTA; and 3) coronary stenosis severity assessed by ICA in percentages, where ≥ 50% stenosis was considered as CAD.

#### 2.4.1 Coronary computed tomography angiography

All CCTA scans were performed using a 256-slice multi-detector CT scanner (Philips Brilliance iCT) following a standardised institutional protocol, with Nexus used for post-processing image enhancement. Pre-procedural evaluation included assessment of renal function (serum creatinine and estimated glomerular filtration rate) to ensure eligibility for iodinated contrast administration. Patients were instructed to fast for 4–6 hours before the scan, and written informed consent was obtained.

To optimize heart rate control, patients with a resting heart rate greater than 70 beats per minute (bpm) were prescribed a single dose of oral bisoprolol (Concor) the night before the procedure. On the day of the procedure, vital signs, including blood pressure and heart rate, were re-evaluated. An 18-gauge intravenous cannula was inserted into the antecubital vein for contrast administration. Patients were positioned feet first and supine on the scanner table, and continuous ECG monitoring was applied using surface electrodes. Sublingual nitroglycerin 0.4 milligrams (mg) was administered immediately prior to imaging to promote coronary vasodilation.

A non-contrast scout scan was obtained to localise the cardiac silhouette and minimize unnecessary radiation exposure. A subsequent non-contrast prospective ECG-gated CT scan (120 kVp, 80–100 mAs, 2.5 mm slice thickness) was performed to evaluate coronary artery calcification using the Xelis calcium scoring software. The Agatston score was automatically computed based on calcified plaque volumes in the LAD, RCA, and LCx arteries. Patients with a CAC score >400 were evaluated cautiously due to limitations in luminal visualization during contrast-enhanced imaging.

For contrast-enhanced CCTA, a bolus-tracking technique was employed. A locator scan (1 axial slice) was placed at the level of the descending aorta, and 90 mL of iodinated contrast was injected at 6.5 mL/s using a power injector. Image acquisition was initiated automatically once a contrast density threshold of 120 Hounsfield Units was detected in the descending aorta. Retrospective ECG gating with dose modulation was used to allow multi-phase image reconstruction. Images were acquired during a single breath-hold and reconstructed at 45%, 75%, and 80% phases of the cardiac cycle using iterative reconstruction algorithms (iDose) to optimise image quality. Radiation exposure was monitored, with the Dose Length Product (DLP) recorded for each scan.

Following scan completion, patients were monitored briefly for post-procedural stability and discharged once clinically appropriate.

#### 2.4.2 Coronary artery calcium score

CAC scores were assessed using non-contrast CCTA and quantified according to the Agatston scoring system. Coronary artery calcification was categorized based on score severity into: 1) no calcification (CAC score = 0); 2) mild calcification (CAC 1–399); and 3) moderate–severe calcification (CAC ≥ 400). The CAC score was automatically calculated using dedicated post-processing software (Xelis Calcium Scoring) based on calcium volume and density within the left main (LM), left anterior descending (LAD), left circumflex (LCx), and right coronary arteries (RCA).

#### 2.4.3 Image post-processing

Scoring was performed on a per-vessel basis, and the total Agatston score was derived by summing the individual vessel scores. All analyses were conducted using a fully automated workflow (Xelis Calcium Scoring, version 2.5.1, INFINITT Healthcare). A board-certified radiologist with over 15 years of experience in cardiac CT interpretation independently reviewed all cases.

All clinical, imaging, and procedural data were retrieved from structured EMRs. CCTA and CAC scoring were interpreted by experienced radiologists, following standardized institutional protocols. Data on CCTA, CAC scoring, and ICA findings were systematically extracted and recorded for analysis.

#### 2.4.4 Invasive coronary angiography

ICA procedures were performed in a dedicated catheterization laboratory by experienced, UK-trained interventional cardiologists, each with over 10 years of clinical experience. Pre-procedural assessment included renal function and clinical stability evaluation, and written informed consent was obtained. Patients were instructed to fast for 6–8 hours prior to the procedure.

Upon arrival in the catheterization laboratory, patients were positioned in the supine position, and continuous ECG monitoring was established. A large-bore 18-gauge intravenous cannula was inserted for fluid and medication administration. Intravenous 3000 international units (IU) of heparin were administered for anticoagulation, and beta-blockers were used when necessary to control the heart rate.

Procedural sedation was achieved with intravenous midazolam and 50 micrograms of fentanyl. The procedure was performed via the right radial artery approach under sterile conditions. After local anesthesia with a local anesthetic (lidocaine), radial artery access was obtained using the Seldinger technique, and a 6 French (6F) sheath was inserted. Intra-arterial 200 mcg of nitroglycerin was administered through the sheath to promote radial artery vasodilation.

Coronary angiography was performed using a 5F Tiger catheter, with visual assessment of stenosis severity: none (0%), mild (25–49%), moderate (50–69%), or severe (≥ 70%) [18]. Patients were monitored briefly and discharged on the same day per radial protocol when stable.

### 2.5 Outcomes

#### 2.5.1 Primary outcomes

The study's primary outcomes focus on evaluating the diagnostic accuracy of CAC scoring as a non-invasive tool compared to the gold standard ICA.

#### 2.5.2 Secondary outcomes

The study's secondary outcomes focus on the accuracy of CCTA imaging in detecting obstructive CAD based on the measurements observed on ICA.

### 2.6 Bias

To minimize selection bias, strict inclusion and exclusion criteria were applied. Measurement bias was addressed by recruiting independent radiologists and cardiologists to review imaging studies in a blinded manner. Observer bias was reduced by engaging an independent biostatistician (J.L.), who conducted the statistical analyses without access to clinical data.

### 2.7 Statistical methods

All statistical analyses were conducted using R version 4.4.0. Descriptive statistics, including means and standard deviations for continuous variables and proportions for categorical variables, were used to summarize baseline characteristics.

The diagnostic performance of CCTA and CAC scoring was evaluated by calculating sensitivity, specificity, positive predictive value (PPV), negative predictive value (NPV), and performing a chi-square test on the three groups of patients with differing CAC scores, with ICA serving as the gold standard.

## 3 Results

### 3.1 Patient selection

**Fig. 1:**
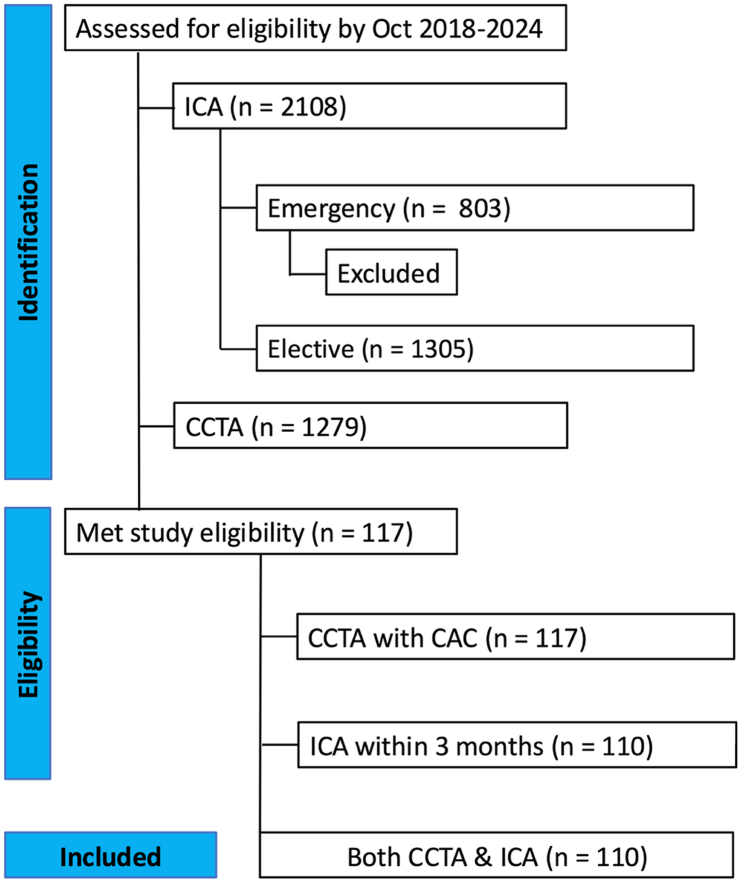
Flowchart demonstrating patient selection for this study.

Between October 2018 and October 2024, 1279 patients underwent CCTA, and 2108 patients underwent ICA at the study center. Of the ICA procedures, 38.1% (n = 803) were performed emergently, while 61.9% (n = 1305) were performed on an elective basis. Among the patients who underwent CCTA, 9.1% (n = 117) also underwent ICA during the study period. However, of these, 91.2% (n = 110) patients met the study eligibility criteria, which required a CCTA with CAC scoring followed by ICA within a maximum of a three-month interval (Fig. 1).

### 3.2 Baseline patient characteristics

**Fig. 2:**
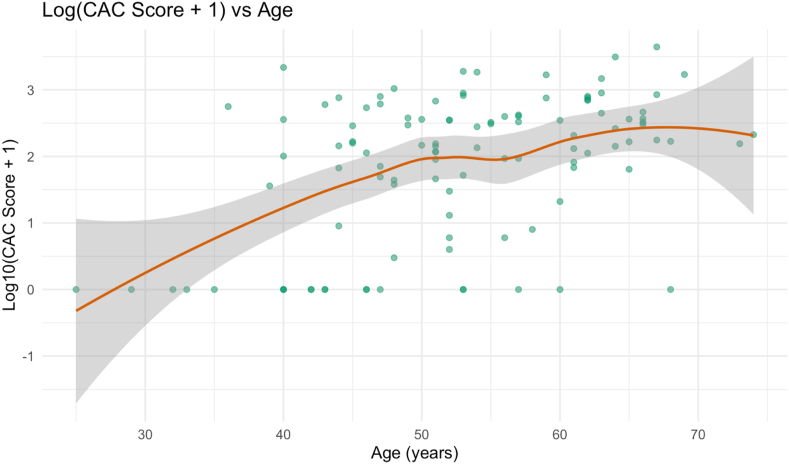
Log-Transformed Coronary Artery Calcium (CAC) Score by Age Scatterplot illustrating the relationship between patient age (in years) and log-transformed coronary artery calcium (CAC) score. A locally estimated scatterplot smoothing (LOESS) curve is overlaid with a 95% confidence interval (shaded area). The trend demonstrates an increase in CAC score with advancing age, with a plateau observed after approximately 60 years of age.

The final cohort in the study included a total of 110 patients who underwent both CCTA, with CAC scoring, followed by an ICA within the dedicated time interval. The cohort comprised 95 males (86.4%) and 15 females (13.6%). The mean age of participants was 53 ± 10 years, with a mean Body Mass Index (BMI) of 27.91 ± 3.66 kg/m^2^. The most prevalent risk factor in the cohort was hyperlipidemia, present in 70.0% of patients, followed by hypertension (37.3%), smoking (21.8%), diabetes mellitus (20.9%), obesity (19.1%), and family history (17.3%). Risk factor prevalence by gender is further detailed in Table 1. A scatterplot of log-transformed CAC score by age is shown in Fig. 2, revealing a positive trend between increasing age and CAC score, with a plateau observed after approximately 60 years of age.

**Table 1:** Baseline patient characteristics.

Study Group Characteristics		
Overall Population (n = 110)		
	**Males (n=95)**	**Females (n=15)**
**Age**	53±9^a^	55 ± 14^a^
**Height**	177.6 ± 7.0^a^	166.3 ± 5.3^a^
**Weight**	87.38 ± 14.82^a^	72.00 ± 14.50^a^
**BMI**	27.62 ± 5.16^a^	27.62 ± 5.16^a^
**ASCVD Risk** **Factor Burden**	1(1–2)^c^	1(1–2)^c^
**Total ASCVD Risk** **Factors**	85.92^b^ (n = 177)	13.59^b^ (n = 28)
**Current Smoking**	13^b^ (n = 23)	3.57^b^ (n = 1)
**Family History of Premature ASCVD**	7.91^b^ (n = 14)	17.86^b^ (n = 5)
**Hyperlipidemia**	37.29^b^ (n = 66)	39.29^b^ (n = 11)
**Hypertension**	20.90^b^ (n = 37)	14.29^b^ (n = 4)
**Obesity**	10.17^b^ (n = 18)	10.71^b^ (n = 3)
**Type 2 Diabetes** **Mellitus**	10.73^b^ (n = 19)	14.29^b^ (n = 4)

a Mean ± standard deviation.

b Percentage (%).

c Median (Interquartile range), BMI: Body Mass Index, ASCVD: Atherosclerotic Cardiovascular Disease.

### 3.3 Diagnostic performance of CAC and CCTA compared to ICA

The diagnostic performance of coronary artery calcium (CAC) scoring and coronary computed tomography angiography (CCTA) was assessed in comparison to invasive coronary angiography (ICA). CAC scoring demonstrated a sensitivity of 91.2%, specificity of 63.2%, positive predictive value (PPV) of 92.2%, and negative predictive value (NPV) of 60%. CCTA demonstrated a sensitivity of 89%, specificity of 57.9%, PPV of 91%, and NPV of 52.4%. While both modalities exhibited high sensitivity and PPV, CAC scoring showed a modest improvement in specificity and NPV over CCTA, though limitations remain in ruling out disease in certain populations (Table 2).

**Table 2:** Diagnostic Performance of CCTA and CAC compared to the gold-standard diagnostic test Invasive Coronary Angiography.

Overall Population (n = 110)				
**Diagnostic Test**	Sensitivity	Specificity	PPV	NPV
**CAC**	91.2^b^	63.2^b^	92.2^b^	60^b^
**CCTA**	89^b^	57.9^b^	91^b^	52.4^b^

This table presents the diagnostic performance of coronary computed tomography angiography scan and coronary artery calcium scoring in the overall population.

^b^Percentage (%), CAC: Coronary Artery Calcium, CCTA: Coronary Computed Tomography Angiography.

### 3.4 CAC score and presence of significant stenosis for all patients

**Fig. 3:**
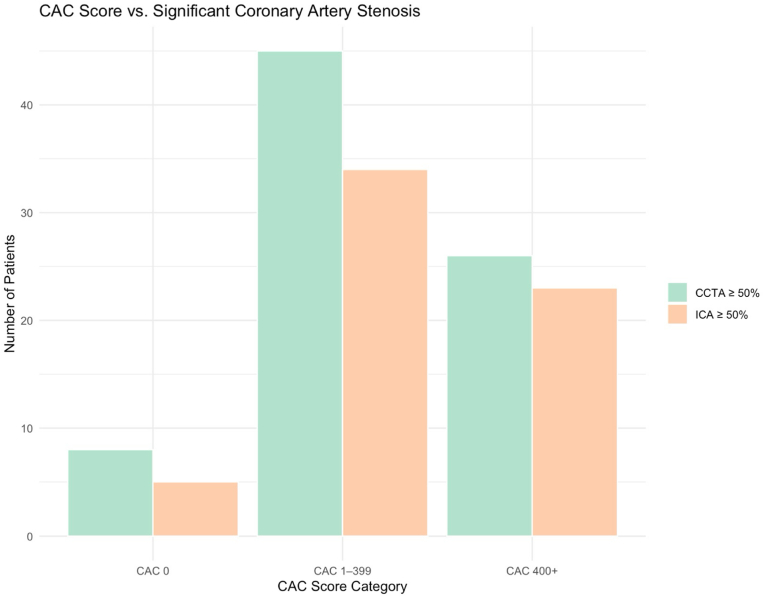
Bar chart showing number of patients with significant stenosis (≥50%) on CCTA and ICA across CAC score categories: CAC 0, CAC 1–399, and CAC ≥400.

The relationship between CAC score category and the presence of significant (≥50%) coronary stenosis, as identified by CCTA and ICA, is displayed in Fig. 3. Among patients with a CAC score of 0 (n = 20), 40% had significant CCTA findings and 25% had significant ICA findings. In the intermediate CAC group (CAC 1–399) (n = 61), 73.8% of patients had CCTA ≥50% stenosis and 55.7% had ICA ≥50% stenosis. In the moderate-to-severe CAC category (CAC ≥400) (n = 29), 89.7% had CCTA ≥50% stenosis and 79.31% (n = 23) had ICA ≥50% stenosis.

### 3.5 Chi-square test analysis of 3 CAC score groups

A chi-square test of independence revealed a statistically significant association between CAC score categories (0, 1–399, and ≥400) and the presence of significant coronary stenosis (≥50%) as determined by ICA (χ^2^ = 14.21, *p* < 0.001), indicating that higher CAC scores are significantly associated with an increased likelihood of CAD (Table 3).

**Table 3:** Association between CAC scores and significant coronary stenosis on ICA.

Overall Population (n = 110)				
**ICA**	CAC = 0	CAC 1-399	CAC≥400	Total
**≥50%**	5 (11.27)^b^ [3.49]^r^	34 (34.38)^b^ [0.00]^r^	23 (16.35)^b^ [2.71]^r^	62
**<50%**	15 (8.73)^b^ [4.51]^r^	27 (26.62)^b^ [0.01]^r^	6 (12.65)^b^ [3.50]^r^	48

^b^Percentage (%).

^r^Pearson residual (r) indicating deviation from expected values; residuals >2 or < -2 are considered statistically significant, ICA: Invasive Coronary Angiography, CAC: Coronary Artery Calcium.

## 4 Discussion

Our study highlights a critical limitation of coronary artery calcium (CAC) scoring. While high CAC scores (≥400) strongly predicted obstructive coronary artery disease (CAD), with 79.3% of this subgroup demonstrating ≥50% stenosis on invasive coronary angiography (ICA), a CAC score of zero did not reliably exclude significant disease. In our cohort, 25% of patients with CAC = 0 had obstructive CAD, challenging the conventional notion that a zero score rules out disease [22]. This underscores the inadequacy of CAC in detecting non-calcified plaque, particularly in younger individuals and those with early-stage atherosclerosis [23]. The significant association between CAC burden and stenosis severity (χ^2^ = 14.21, p < 0.001) confirms its value in risk stratification. However, relying solely on calcification risks missing high-risk, non-calcified plaques that may still precipitate acute coronary syndromes (ACS) [24].

These findings are consistent with prior evidence linking higher CAC scores with CAD. Studies have shown that CAC scores above 100 are significantly associated with obstructive CAD [25], and CAC ≥400 is an independent marker of chronic coronary changes [26]. Others report strong positive correlations between CAC scores and coronary artery stenosis, as well as associations with conventional cardiovascular risk factors [27]. Negative CAC scores have led to significant downward risk reclassification [28], though their reliability is reduced in patients under 40 years due to the increased prevalence of non-calcified plaques [29,30].

Our findings align with studies showing that while CAC has good negative predictive value (NPV), it may be inferior to total or calcified plaque volume measurements in predicting obstructive CAD [31]. Despite high sensitivity (91.2%) and positive predictive value (92.2%), the specificity (63.2%) and NPV (60%) were suboptimal. These results suggest that CAC alone may be insufficient to rule out disease, especially in symptomatic or intermediate-risk individuals.

Recent trials support incorporating plaque morphology assessments to enhance diagnostic accuracy. Coronary computed tomography angiography (CCTA) has shown promise in detecting non-calcified vulnerable plaques [32]. The SCOT-HEART trial demonstrated that CCTA improved cardiovascular risk stratification and outcomes compared to standard care [33]. The PROMISE trial further highlighted that combining CAC scoring with CT-derived fractional flow reserve (FFR-CT) enhanced diagnostic performance for functionally significant lesions [34].

In our cohort, 55.7% of patients with intermediate CAC scores (1–399) had obstructive CAD, compared to 25% in the CAC = 0 group and 79.3% in those with CAC ≥400. This underscores that intermediate scores reflect atherosclerotic burden but cannot reliably exclude significant stenosis. For such patients, individualized assessment should integrate clinical context and may warrant additional imaging. These findings reinforce that CAC = 0 should not automatically rule out further workup in the presence of symptoms or high suspicion.

This study's strengths include strict eligibility criteria, standardized imaging protocols, and blinded outcome assessment. However, limitations include its single-center, retrospective design and modest sample size. A major limitation of CAC scoring is its inability to detect non-calcified plaque (NCP), which can still lead to ACS. Studies show that CAC = 0 does not fully exclude NCP or significant CAD [35]. While the burden of NCP in asymptomatic individuals with CAC = 0 is generally low, high-risk subgroups may harbor occult disease. In such cases, the addition of CCTA may aid risk stratification [36]. However, due to the low prevalence of NCP in CAC = 0, a careful risk-benefit approach is advised before routine CCTA. Future directions include multimodal strategies combining CAC with CCTA and the use of artificial intelligence for plaque characterization [34,37].

Ultimately, coronary risk assessment must evolve beyond a binary interpretation of CAC scores. While CAC remains an efficient and accessible screening tool, it is not a standalone diagnostic solution. Future guidelines should reflect the complex interplay between calcification, plaque vulnerability, and stenosis severity, and encourage approaches that integrate clinical, anatomical, and functional data.

## 5 Conclusions

This study demonstrates that CAC scoring alone is insufficient for ruling out obstructive coronary artery disease. Twenty-five percent of patients with CAC = 0 had significant stenosis on invasive angiography, underscoring its inability to detect non-calcified plaque. While high CAC scores (≥400) were predictive of disease, modest specificity limits their standalone utility. Until multimodal approaches become standardized, clinicians must interpret CAC results cautiously, recognizing that the absence of calcium does not equate to the absence of risk.

## CRediT authorship contribution statement

**Jonathan Mokhtar:** Writing – review & editing, Writing – original draft, Visualization, Methodology, Formal analysis, Data curation, Conceptualization. **Mohammad Albaree:** Writing – review & editing, Writing – original draft, Data curation. **Virginia Battistin:** Writing – original draft, Data curation. **Mohamed Asbaita:** Writing – review & editing, Writing – original draft. **Fatemeh Akbarpoor:** Writing – review & editing, Formal analysis. **Jeyaseelan Lakshmanan:** Software, Formal analysis. **Hassan El-Tamimi:** Supervision, Investigation.

## Data availability statement

The data that support the findings of this study are available from the corresponding author upon reasonable request.

## Funding

This work was supported by the 10.13039/501100020917Mohammed Bin Rashid University of Medicine and Health Sciences (10.13039/501100020917MBRU), Dubai Health, Dubai, United Arab Emirates, through internal institutional funding. The funding source was not involved in the study design, data collection, data analysis, interpretation of the results, or the writing and submission of the manuscript.

## Declaration of competing interest

All authors report no relationships that could be construed as a conflict of interest. All authors take responsibility for all aspects of the reliability and freedom from bias of the data presented and their discussed interpretation.
